# Microneedles mediated-dermal delivery of Vitamin C: Formulation, characterization, cytotoxicity, and enhancement of stability

**DOI:** 10.1016/j.heliyon.2024.e37381

**Published:** 2024-09-03

**Authors:** Rania Hamed, Amani D. AbuKwiak, Rafa Aburayya, Ahlam Zaid Alkilani, Lama Hamadneh, Mais Naser, Yasmeen Al-Adhami, Ala A. Alhusban

**Affiliations:** a Department of Pharmacy, Faculty of Pharmacy, Al-Zaytoonah University of Jordan, Jordan, Amman, 11733, Jordan; b Department of Pharmacy, Faculty of Pharmacy, Zarqa University, Zarqa, Zarqa, 13110, Jordan; c Department of Basic Medical Sciences, Faculty of Medicine, Al-Balqa Applied University, P.O. Box 206, Al-Salt, 19117, Jordan

**Keywords:** Microneedles, Polymeric solutions, Vitamin C, Skin delivery, Stability, Stabilizing agents

## Abstract

Vitamin C (VIT C) is an antioxidant that prevents skin aging. Although dermal delivery is one of the most effective routes to transport VIT C to the skin, the impact of this route can be limited by the barrier function of the *stratum corneum* (SC). Additionally, VIT C rapidly oxidized and degraded under light and temperature. Therefore, this study provides an approach to utilizing microneedles (MNs) to improve the dermal delivery of VIT C and enhance its stability by incorporating a stabilizing system of ethylenediaminetetraacetic acid (EDTA) and sodium metabisulfite (Meta) within the MNs. Vitamin C microneedles (VIT C MNs) were fabricated using different biodegradable polymers and various concentrations of EDTA/Meta. VIT C MNs were evaluated for morphology, VIT C content, mechanical properties, dissolution rate, needles’ insertion, physicochemical properties, *ex vivo* permeation, viscosity of VIT C polymeric solutions, cytotoxicity, and stability. The results showed that VIT C MNs were uniform and mechanically strong. The recovery of VIT C in MNs was 88.3–90.0 %. The dissolution rate of MNs was <30 min. The flux of VIT C varied based on the composition of MNs. VIT C MNs demonstrated safety against human dermal fibroblasts. VIT C MNs with EDTA/Meta (0.1/0.3 %) were stable under different storage conditions for two months. In conclusion, VIT C MNs were successfully developed using biodegradable polymers, and the stabilizing system (EDTA/META) provided a stable dermal delivery system for VIT C to protect skin from aging.

## Graphical abstract

**Figure:**
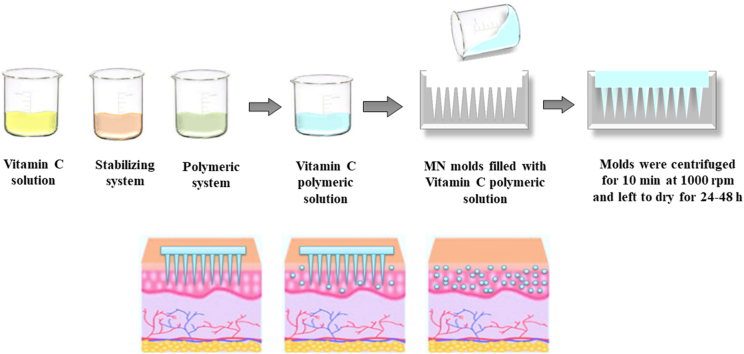

## 1 Introduction

All living organisms undergo slow biological aging, characterized by several irreversible cellular and physiological changes [1]. The formation of reactive oxygen species (ROS), which causes oxidative stress and severe cellular damage in various cells and tissues, is one of the essential aspects of aging that significantly accelerates the aging process [2]. Vitamin C (VIT C) is an antioxidant agent that acts as a free radical scavenger, protecting lipids, DNA, and proteins from oxidative damage [3]. It is essential for reducing the effects of oxidative stress and possibly slowing down the aging process [4]. The level of VIT C is high in healthy skin, where it performs important and well-known roles, such as encouraging collagen formation and functioning as an antioxidant shield against UV-induced photoaging [5]. Notably, the epidermal layer contains a significantly higher concentration of VIT C than the dermal layer [5].

VIT C is frequently used for cosmetic purposes to moisturize the skin, enhance the tone and texture, and lessen aging. The physical and chemical characteristics of VIT C are determined by its chemical structure, where this organic acid is weak, water-soluble, and rapidly oxidized or destroyed by light [6]. It is a highly unstable vitamin that oxidizes when exposed to water-based solutions [7]. In addition, it is widely used as an anti-aging cosmetic ingredient, commonly in the form of sodium ascorbate or ascorbyl palmitate as derivatives of VIT C [8]. The oral administration of VIT C is insufficient for delivering this compound effectively to the skin [9]. The only practical way to deliver VIT C to the skin is through the dermal or local route, which may induce collagen production and slow aging [6]. The barrier function of the *stratum corneum* (SC) affects the absorption of VIT C from the skin, even though topical application appears to be an efficient route for delivering VIT C to skin layers [10]. Moreover, the stability and efficacy of VIT C in topical preparations are an obstacle since it is easily oxidized in cosmetic formulations [11,12].

To maintain the biological activity of VIT C and effectively delay its degradation via oxidation, sodium metabisulfite (Meta) was frequently used as an antioxidant [13]. Additionally, it was found that the chelating agent disodium salt of ethylenediaminetetraacetic acid (EDTA) exhibits a synergistic stabilizing activity of VIT C [12]. Moreover, it has been shown that the penetration of the drugs through the SC can be improved by the application of chemical (passive) and physical (active) techniques, either individually or in combination. In chemical techniques, chemical additives such as chemical penetration enhancers and nanocarriers can passively facilitate the delivery of drug molecules through the SC. Physical methods such as iontophoresis, phonophoresis, electroporation, thermal ablation, nongravitational and cavitational ultrasound, microdermabrasion, and microneedles utilize external energy or physically disrupt the SC [14].

Microneedles (MNs) have been utilized to improve the transcending of VIT C through the skin barrier via temporal noninvasive disruption, where the passive delivery of VIT C might be inadequate due to skin complexity [4,9,15]. Studies have investigated the efficacy of VIT C gel when applied together with polymeric MNs against skin redness and irritation [16], in addition to a significant boost in the transdermal delivery of VIT C when compared to topical solution [17]. The use of MNs offers potential advantages, including controlled-release behavior, reduced side effects, and less frequent dosing [18]. Additionally, MNs can load drugs, proteins, and hormones sensitive to degradation without loss of activity [19,20].

Limited research has explored the use of dissolving MNs for the dermal delivery of VIT C [[21], [22], [23]]. Lee et al. [21] successfully developed dissolving MNs using hyaluronic acid, demonstrating their effectiveness in reducing skin wrinkles without irritating. Additionally, Park et al. [22] developed VIT C dissolving MNs using the biocompatible polymer carboxymethyl cellulose. These MNs showed a six-fold enhancement in skin permeability and antioxidant activity of VIT C compared to the topical application of VIT C alone. Nevertheless, these studies did not evaluate the thermal stability of VIT C loaded within the MNs, where the instability of VIT C remains a major concern. A recent study by Leelawattanachai J. et al. [23] investigated the use of cationic polymer polyethyleneimine (PEI) to improve the stability of VIT C loaded into dissolving MNs. The stability results found that PEI could significantly protect VIT C from degradation within MNs after storage for at least 2 months at all the tested temperatures (−20, 4, 25, and 40 °C).

Therefore, this study aims to develop polymeric dissolving MNs as a minimally invasive approach for administering VIT C into the dermal layer of the skin and enhancing its stability, mainly for cosmetic applications. The rationale for developing VIT C dissolving MNs is that these MNs would enhance the penetration of VIT C through the skin and improve its stability by incorporating a stabilizing system of META and EDTA within the MNs. MNs form micropores in the skin surface that release VIT C from the dissolving matrix and transport it into the dermal region with minimally invasive and painless administration. VIT C MNs were fabricated using biodegradable polymers such as polyvinyl alcohol (PVA) and polyvinyl pyrrolidone (PVP). Additionally, a combination of EDTA and Meta (EDTA/Meta), which provides a synergistic stabilizing effect for VIT C, was incorporated into the MNs. VIT C MNs were characterized in terms of morphology, content of VIT C, mechanical properties, dissolution rate, needle insertion, invasiveness of MNs in rat skin, differential scanning calorimetry (DSC), Fourier-transform infrared (FTIR), *ex vivo* permeation, viscosity, cytotoxicity, and short-term stability.

## 2 Materials and methods

### 2.1 Materials

Vitamin C (VIT C, L-ascorbic), polyvinyl alcohol 87–90 % hydrolyzed (PVA, average molecular weight (MW) 13,000–20,000), and PVA 5–88 EMPROVE® (PVA 5–88, average MW 67,000) were purchased from Sigma-Aldrich (Dorset, UK). Polyvinyl pyrrolidone (PVP, average MW 40,000) was purchased from Ashland (Kidderminster, UK). Sodium metabisulfite (Meta) and ethylenediaminetetraacetic acid disodium salt (EDTA) were purchased from Alfa Aesar (Haverhill, MA, USA). Phosphate buffered saline (PBS, pH 6.8) and MTT (3- (4, 5-dimethylthiazol-2-yl) 2, 5-diphenyl tetrazolium bromide) assay reagent were purchased from Sigma Aldrich® (Dorset, UK). MN molds (Silicone Template ST-06) were purchased from Micropoint Technologies PTE LTD (Singapore). Ultrapure water (UPW) was used in all experiments. All other reagents were of analytical grade and used as received.

### 2.2 Methods

#### 2.2.1 Fabrication of VIT C MNs

Dissolving MNs loaded with VIT C, labeled F1 through F6, were fabricated using PVP and PVA polymeric solutions (Table 1). The polymers were dispersed in ultrapure water (UPW) and stirred for 2 h on a hot plate magnetic stirrer set at 200 rpm and 90 °C. After that, 300 mg of VIT C was dissolved in 1 mL of UPW to make the VIT C solution. To improve stability, different quantities of EDTA and Meta (EDTA/Meta), used as stabilizing agents, of 0.1/0.3, 0.2/0.6, and 0.3/0.9 % were added to the VIT C solution and then to the aqueous polymer solution with continuous stirring at 200 rpm until a clear homogenized solution was formed. The mixtures (150 mg) were poured into the MN molds and centrifuged for 10 min at 1000 rpm at 25 °C using a centrifuge (HERMLE Z 366 K; Germany). After drying for 48 h at 25 °C, a backing layer composed of 30 % PVA 5–88 and 10 % PVP at a ratio of 7:3, respectively, was added to cover the MNs and facilitate their detachment from the mold. Afterward, MNs were carefully removed from the mold.

**Table 1:** Composition of VIT C MNs prepared using different concentrations and ratios of biodegradable polymers (PVA, PVP, PVA 5–88) and EDTA and Meta stabilizing agents.

**VIT C** **MNs**	**MNs matrix**				**Backing layer**			**Stabilizing agents**					
								**Group 1**		**Group 2**		**Group 3**	
	**VIT C 30 % w/v (mL)**	**PVA 15 % (mL)**	**PVP 40 % (mL)**	**PVA 20 % (mL)**	**VIT C 30 % (mL)**	**PVP 10 % (mL)**	**PVA 5–88 30 % (mL)**	**EDTA/Meta (%)**					
F1	1	1	–	–	–	7	3	0.1	0.3	0.2	0.6	0.3	0.9
F2	1	–	1	–	–	7	3	0.1	0.3	0.2	0.6	0.3	0.9
F3	1	2	1	–	–	7	3	0.1	0.3	0.2	0.6	0.3	0.9
F4	1	–	–	1	–	7	3	0.1	0.3	0.2	0.6	0.3	0.9
F5	1	1	1	–	–	7	3	0.1	0.3	0.2	0.6	0.3	0.9
F6	1	–	1	1	–	7	3	0.1	0.3	0.2	0.6	0.3	0.9

#### 2.2.2 Characterization of VIT C MNs

##### 2.2.2.1 Morphology

The dimensions (width and height) and appearance of VIT C MNs were visualized under a light microscope (Leica Microscope, Milton Keynes, UK) at 10x magnification and a USB digital microscope (Skybasic 50X-1000X) connected to a computer. Sharp and intact MNs were only used for further characterization.

##### 2.2.2.2 Determination of VIT C content in MNs

To determine the amount of VIT C in MNs, one MN array of F1, F2, and F3 MNs was placed in a 10 mL-volumetric flask, dispersed in 10 mL UPW, and sonicated for 15–20 min. F1 and F2 were loaded with 22.5 mg of VIT C in 150 mg polymeric solutions, whereas F3 was loaded with 11.25 mg of VIT C in 150 mg polymeric solution. These polymeric solutions were then placed into the 11 x 11 needle array silicon molds to prepare the MN patches. A calibration curve of VIT C was built to quantify the amount of VIT C in each MN array using a UV–vis spectrophotometer. A standard stock solution was prepared by dissolving 30 mg of VIT C in 10 mL UPW and then diluted to a concentration range of 3.125–50 μg/mL. The recovered concentration of VIT C in the loaded MNs was determined using a calibration curve of VIT C. The absorbance of the sample solutions was measured at 266 nm. The amount of VIT C loaded into MNs was assessed in triplicate, and the mean and standard deviation (SD) were reported.

##### 2.2.2.3 Mechanical properties

The mechanical properties of VIT C MNs were assessed using a Texture Analyzer (TA.XT2) (Stable Microsystems, Haslemere, UK) to evaluate the force tolerance for each MN upon applying a certain force. Each MN was compressed at a target force of 32 N/array for 30 s [24]. The applied force of 32 N/array for 30 s was chosen to simulate the force of the thumb [25,26]. This ensures that MNs can penetrate the skin effectively without causing discomfort to the user. The following paragraph has been added to the revised manuscript. MNs were placed on TA.XT2 stainless steel flat plate with needles pointing upwards. Once the targeted force had been applied to the MN arrays, the probe was raised upward and pushed away from the MNs at a speed of 1 mm/s. Each MN array was observed using a light microscope (at 10x magnification) before and after compression to determine the percentage of height reduction (%Height reduction), reflecting the average height and dimension change due to force application. The %Height reduction for MNs was evaluated in triplicate and calculated according to the following equation [27]:

$$\%\text{Height}\;\text{reduction}=\frac{\left(Height\;before\;applying\;force\;-\;Height\;after\;applying\;force\right)\;}{Height\;before\;applying\;force}\times 100$$

##### 2.2.2.4 Dissolution rate

Skin samples were prepared as described [28]. Briefly, male Wistar rats of 180–200 g weight were maintained in the animal care center of the Faculty of Pharmacy, Al-Zaytoonah University of Jordan, Jordan, and used in the experiment. The experiment was performed with the approval of the Research Ethics Committee at Al-Zaytoonah University of Jordan (IRB No. 01/10/2022–2023). The rats were anesthetized with diethyl ether and sacrificed via cervical dislocation. Pieces of full-thickness rat skin were used, wrapped in aluminum foil, and kept at −80 °C until used. Dissolution studies were conducted as described in Ref. [29]. Before the study, the skin was soaked and cleaned in phosphate-buffered saline (PBS, pH 7.4) for 15 min before use. The dermal side of full-thickness rat skin was used to place MNs manually by the thumb for 1 min, after which the MNs were detached at predetermined time intervals (5, 10, 20, and 30 min) and were immediately observed under a light microscope at 10x magnification to evaluate the dissolution rate of needles after each time interval.

##### 2.2.2.5 Insertion studies

The insertion of MNs was assessed using the artificial flexible thermoplastic membrane Parafilm M (Bemis Company Inc., Soignies, Belgium), which simulates skin penetration as described in Ref. [26]. Eight layers of Parafilm M were folded to a thickness of ∼1 mm. MNs were attached to the moveable probe of the Texture Analyzer (TA.XT2), and the probe was lowered onto the folded Parafilm M at a speed of 1.19 mm/s until the targeted force of 32 N/array was obtained. The number of layers perforated and the holes made in each layer of Parafilm M were determined using a light microscope at 10x magnification.

##### 2.2.2.6 Differential scanning calorimetry

The thermal behavior of pure VIT C, PVA, PVP, and PVA 5–88, physical mixture (PM) of MNs, and VIT C MNs were examined using a differential scanning calorimetry (DSC, Netzsch, Germany). A sample of 3 mg was placed in the DSC aluminum crucible, sealed, and scanned at a temperature range of 30 to 300 °C and a flow rate of 10 mL/min. An extra empty aluminum pan was sealed and used as a reference control.

##### **2.2.2.7** **Attenuated total reflectance-Fourier transform infrared** (**ATR-FTIR)**

The ATR-FTIR analysis was conducted to investigate the compatibility among MN components using PerkinElmer UATR-II (Llantrisant, UK). The ATR-FTIR analysis was performed for pure VIT C, PVP, PVA, and PVA 5–88, physical mixture (PM) of MNs, and VIT C MNs. The spectra were obtained using 32 scans at a resolution of 2 cm^−1^ over a frequency range of 4000 to 400 cm⁻^1^. The analysis of the spectral data was performed using Spectragryph Version 1.2.16.1.

##### 2.2.2.8 *Ex vivo* permeation

The *ex vivo* permeation of VIT C MNs was assessed using Franz diffusion cells of 25 mm aperture diameter and 1.76 cm^2^ diffusion surface area (Copley Scientific Ltd., Nottingham, UK). The *ex vivo* permeation experiment for VIT C aqueous solution was not conducted due to the instability of VIT C in aqueous systems [30]. The experiment was carried out at 32 °C. Full-thickness rat skin was taken from the back of a rat, according to the approved protocols for rat skin preparation by the Ethics Committee for Scientific Research, approval number (IRB No. 01/10/2022–2023). The SC was in contact with the donor phase, and the rat skin was clamped between the donor and receptor compartments. The receptor compartment was filled with phosphate-buffered saline (PBS, pH 7.4) and stirred at 600 rpm. VIT C MNs were manually pressed for 30 s into rat skin and placed in the donor compartment. Subsequently, samples of 1 mL were withdrawn from the receptor compartment every 30 min for 4 h and replaced by 1 mL of fresh PBS following each sample collection. A UV–vis spectrophotometer at 266 nm was used to determine the amount of VIT C in the samples. The amount of VIT C loaded within MNs was considered the amount in the donor compartment used to calculate the permeation parameters. The amounts of VIT C loaded in F1-F3 MNs were 21713.24 ± 67.61, 21450.21 ± 432.22, and 11043.70 ± 300.40 μg, respectively. The cumulative amount of VIT C permeated through skin per unit surface area (Q/A) was plotted versus time (t) to calculate the steady-state flux (J_ss_, μg/cm^2^/hr) from the slope of the linear portion of the (Q/A) versus (t) plot. Moreover, the permeability coefficient (P, cm/h) was calculated according to the below equation [31]:

P = J_ss_/C_0_

where,

P: Permeability coefficient (cm/h)

J_ss_: The steady-state flux (μg/cm^2^/h)

C_0_: The initial VIT C concentration loaded into the MNs and placed in the donor compartment.

##### 2.2.2.9 Mechanism of release

The mechanism of VIT C release from MNs was investigated by fitting the *ex vivo* skin-permeation profiles into zero-order, first-order, Higuchi, and Korsmeyer-Peppas models.

##### 2.2.2.10 Viscosity study

The viscosity of VIT C polymeric solutions was determined at 25 ± 0.5 °C using a rotational rheometer (QC Rheolab, Anton Paar, Graz, Austria) with the C-CC27/SS Coaxial Cylinder System over a shear rate of 10–1000 s^−1^ with 30 measuring points. The duration of the measuring point was 1 s, and the total interval duration was 914.2 s.

##### 2.2.2.11 Cytotoxicity study

The cytotoxicity of VIT C MNs was assessed using the MTT assay method and human dermal fibroblast cells (CCD-1064SK) as described in Ref. [32]. The cells were cultured in a 5 % CO_2_ incubator at 37 °C with 89 % Dulbecco's Modified Eagle's Medium (DMEM, Sigma Chemical Co., serial number D5648), 10 % fetal calf serum (FCS, Gemini Bioproducts, Cat. No. 100–106), and 1 % penicillin-streptomycin-neomycin solution. In 96-well surface-treated plates, the cells were seeded at a density of 10,000 cells per well and left to adhere to the surface for 24 h. The following day, a volume of 20 μL of various serial dilutions (100, 50, 25, 12.5, 6.25, 3.12, and 1.56 μM) of VIT C polymeric solution containing stabilizing agents EDTA/Meta, VIT C solution, and blank polymeric solution were added to the appropriate wells and incubated for 72 h at 37 °C. After treatment, a volume of 20 μL of 5 mg/mL solution (4,5-dimethylthiazol-2-yl)-2,5-diphenyltetrazolium bromide (MTT) tetrazolium substrate was added to each well and incubated for 4 h at 37 °C, then MTT-containing media was discarded. To assess the absorbance of the samples, the plates were analyzed using a microplate reader (SYNERGY-BioTek) at 450 and 570 nm.

##### 2.2.2.12 Stability studies

VIT C MNs were stored refrigerated (4 °C) and at room temperature (20 °C) for 2 months. Morphology, mechanical properties, VIT C content, and dissolution rate for VIT C MNs with and without stabilizing agents (control group) were evaluated to select the ideal concentration of EDTA/Meta used to stabilize VIT C within MNs under different storage conditions.

#### 2.2.3 Statistical analysis

One analysis of variance (ANOVA) test was utilized to calculate the statistical significance of the viscosity curves of VIT C polymeric solutions using GraphPad Prism 5 software. The level of significance was accepted at *p* < 0.05.

## 3 Results and discussion

### 3.1 Morphology of VIT C MNs

**Fig. 1:**
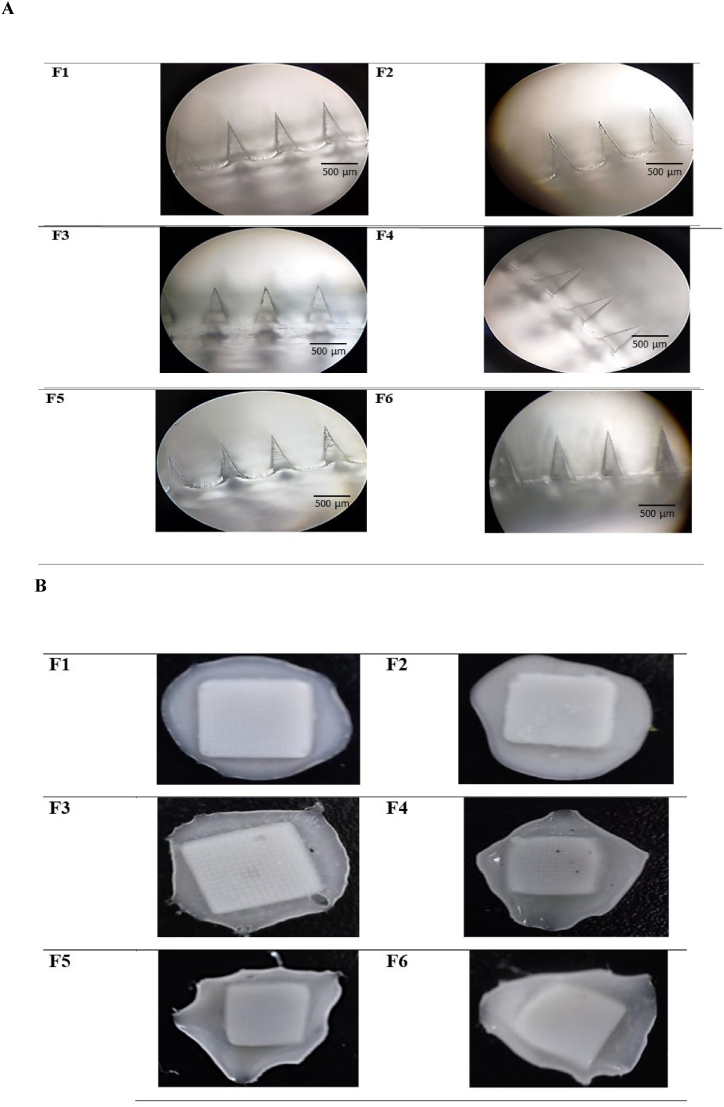
**(A)** Morphology of VIT C MNs (F1-F6) observed under a light microscope at10x magnification and **(B)** Images of VIT C MNs (F1-F6) with 0.3:0.9 % EDTA/Meta of opaque white color, observed under a USB digital microscope (Skybasic 50X-1000X) connected to a computer.

VIT C MNs (F1-F6) were detached from the molds and showed a complete array of needles with sharp pyramidal tip shapes when observed under a light microscope (10x magnification) (Fig. 1A). MNs were 601.5 ± 7.4 μm in height and 252 ± 2.0 μm in width, with MN baseplates that were strong enough to be easily removed from the molds without causing damage to the arrays. The use of 0.1/0.3 and 0.2/0.6 % EDTA/Meta maintained the morphological shape of F1-F6 MNs. However, it was found that using EDTA/Meta at the highest concentration (0.3/0.9 %) decreased the transparency of F1-F6 MNs, appearing opaque white (Fig. 1B). Thus, F1-F6 MNs with 0.3/0.9 % EDTA/Meta were excluded from further studies.

### 3.2 Determination of VIT C content in MNs

A calibration curve of VIT C at a concentration range of 3.125–50 μg/mL with a correlation coefficient (R^2^) of 0.9992 was used to determine the amount of VIT C recovered from F1-F6 MNs. The recovery of VIT C from F1-F3 MNs was 98.85 ± 0.04, 88.33 ± 0.03, and 89.10 ± 0.04 %, respectively. The percentages reported (89.10–98.85 % for F1-F3 MNs) reflect the recovery of VIT C in the microneedle array, not the proportion of VIT C in the formulation relative to other components like PVA. To clarify the drug loading, F1, F2, and F3 MNs were loaded with 22.24, 19.87, and 10.02 mg of VIT C, respectively, per microneedle array. The formulation was poured into silicone molds containing 121 needles arranged in an 11 × 11 formation to make 121 needles with dimensions of 600 μm in height, 300 μm in width, and 300 μm in interspacing. F4-F6 showed a lower VIT C content of 65.04 ± 0.04, 36.61 ± 0.11, and 57.01 ± 0.07 %, respectively, indicating that the polymeric matrix had insufficient capacity to incorporate VIT C within MNs. Therefore, F4-F6 MNs were excluded from further characterization.

### 3.3 Mechanical properties of VIT C MNs

MNs need to be strong enough to tolerate the force that simulates the manual tapping by the human thumb to perform temporal pores into skin layers and deliver the exact amount of loaded compound to the skin [33]. The morphological changes in MN height (%Height reduction) and shape before and after applying a force of 32 N/array for 30 s are illustrated in Table 2. The needles of F1-F3 showed a %Height reduction of <10 %, indicating that they could tolerate the pressure applied by the thumb and resist the compression force [9]. F1-F3 showed a %Height reduction of 8.1, 6.4, and 7.2 %, respectively, confirming that MNs were mechanically strong without fracture. Studies have shown that MNs fabricated using PVA, PVP, or a combination of PVP and PVA exhibit tolerable mechanical resistance and hardness [34,35], particularly when polymers are used at high concentrations or with high molecular weights in the polymeric mixture [36,37].

**Table 2:** Morphological changes and %Height reduction in F1-F3 MNs before and after applying force (32 N/array for 30 s) visualized under a light microscope (10x magnification). Data is presented as mean ± SD (n = 3).

VIT C MNs	Microscopic images		Height before (μm) ± SD	Height after (μm) ± SD	%Height reduction
	Before compression	After compression			
F1			590.8 ± 25.3	542.8 ± 54.7	8.1
F2			595.1 ± 29.7	554.6 ± 55.0	6.4
F3			593.4 ± 32.1	550.9 ± 24.9	7.2

### 3.4 Dissolution rate of VIT C MNs

**Fig. 2:**
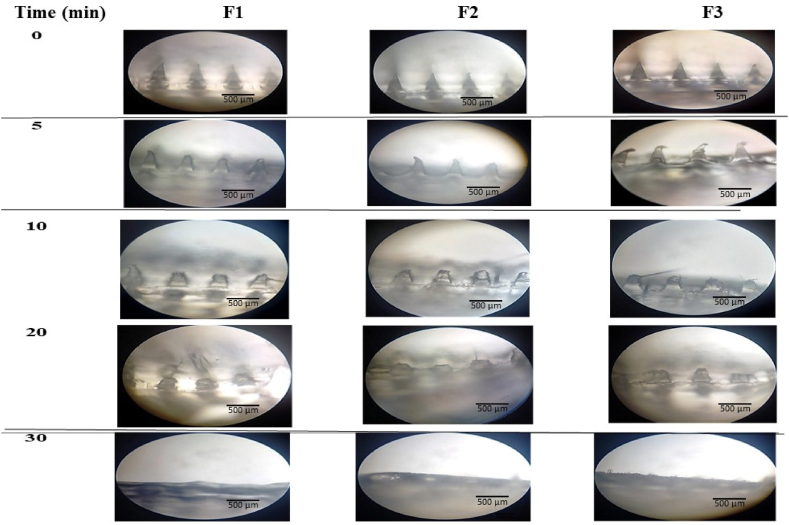
The dissolution rate of F1-F3 MNs following insertion through rat skin displayed after 5, 10, 20, and 30 min time intervals, observed under a light microscope at 10x magnification.

F1-F3 MNs were visualized under a light microscope (10x magnification) to determine their dissolution at pre-determined time intervals. At zero time, F1-F3 MNs were intact. After 5 min of post-insertion into rat skin, F1-F3 MNs start dissolving. After 10 min post-insertion, F1-F3 were dissolved, showing a %Height reduction of >50 %. The dissolution of F1-F3 was completed after 20–30 min (Fig. 2). Despite the mechanical tolerance, F1-F3 exhibited fast dissolution rates of <30 min. The fast dissolution rate of VIT C MNs is attributed to the fact that F1-F3 are formulated with the water-soluble polymers PVP and PVA [38]. When MNs are applied to the skin at ambient temperature, PVP and PVA absorb the surrounding interstitial fluid, and needles dissolve [38,39], requiring 20–30 min to dissolve completely. The dissolution of MNs in the skin can prevent tissue damage caused by the mechanical force applied during application without leaving sharp needles behind [39,40]. A balance between the rapid dissolution rate of MNs and the mechanical strength of the polymer(s) used to formulate MNs is vital for the successful penetration of the MNs in the skin and their detachment from the base [39].

The hydrogen bonds, responsible for the physical crosslinking between PVA and PVP, provide mechanical strength to the MNs [41]. These relatively weak non-covalent interactions increase the free volume within the MNs matrix. When the highly water-soluble polymers PVA and PVP come in contact with the interstitial fluid of the skin, water molecules interact with the hydrogen bonding sites, disrupting these bonds and leading to rapid swelling and dissolution of the MNs within 30 min [42].

The dissolution rate of VIT C MNs, between 20 and 30 min, is in agreement with previous studies [37,43,44]. For instance, it has been shown that lidocaine-dissolving MNs formulated with 25 % PVP dissolved in the skin after 20 min [43]. Additionally, alendronate sodium dissolving MNs formulated with different ratios of 30 % PVP and 15 % PVA completely dissolved after 15–20 min [44]. Moreover, Donnelly et al. [37] showed that itraconazole nanocrystal-loaded dissolving MNs, formulated with a combination of 15 % PVA and 25 % PVP, dissolved in the skin after 30 min of application.

### 3.5 Insertion of VIT C MNs

**Fig. 3:**
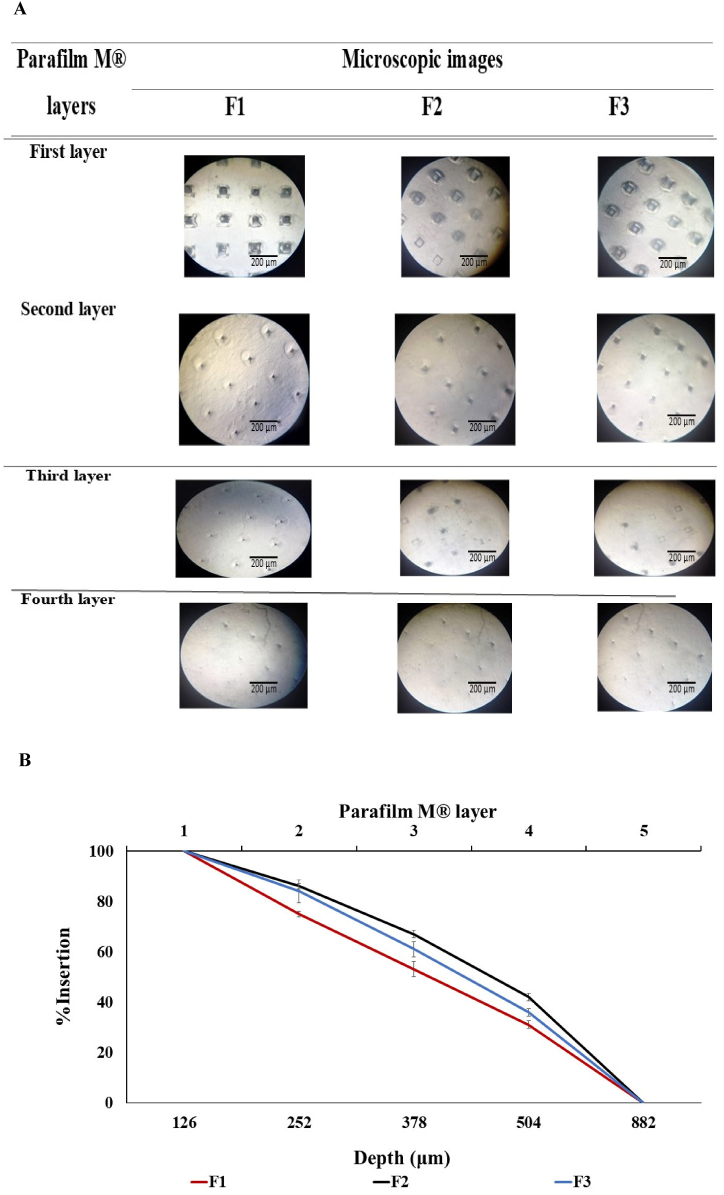
**(A)** Needles insertion of F1-F3 MNs through the first four layers of Parafilm M® as observed under a light microscope at 10x magnification and (**B)** Relationship between three variables: the depth of Parafilm M®, number of Parafilm M® layers, and %Insertion of F1-F3 MNs. Data are presented as mean ± SD (n = 3).

The insertion of F1-F3 MNs was visualized under a light microscope (10x magnification). F1-F3 MNs penetrated the first four layers of Parafilm M without being fractured, corresponding to a depth of 378–504 μm, where each layer has a thickness of 126 μm [26] (Fig. 3A). This demonstrates the ability of MNs to reach the SC and epidermis with a thickness of 10–20 and 100–150 μm, respectively [26,45]. F1-F3 had a %Insertion of 98–100 % in the first layer. Then, the %Insertion decreased in the subsequent layers, attaining 31, 42, and 36 % in the fourth layer of F1-F3, respectively (Fig. 3B). This agrees with the mechanical strength results of F2> F3> F1, where F1 displayed the highest height reduction of 8.1 %, which may be associated with the least penetration through the deepest layers. Fig. 3B illustrates the relationship between the %Insertion in the first four layers, the depth of Parafilm M layers ranging between 378 and 504 μm, and the number of Parafilm M layers perforated by F1-F3. The insertion of MNs into Parafilm M is attributed to the polymeric matrix of PVA and PVP, which can provide MNs flexibility, elasticity, and hardness [46,47]. PVP has a distinctive rigid chemical structure with rings and hydrogen bonds that strengthen the MNs (F2) [48,49]. Moreover, the effect of the PVA/PVP combination in improving the mechanical properties of MNs, particularly F3, has been reported in Ref. [50]. Although PVA and PVP, used in the MNs’ fabrication, can form abundant intramolecular hydrogen bonds within their chains, they can also form intermolecular hydrogen bonds with each other [51,52]. The hydrogen bond interactions between PVA and PVP lead to the physical crosslinking between these two polymers, owing to the interchain hydrogen bonding between the hydroxyl and carbonyl groups of PVA and PVP, respectively [53]. The specific arrangement of these groups and the presence of multiple hydrogen bonding sites in PVA and PVP allow for abundant intra- and intermolecular hydrogen bond interactions between the two polymers, further increasing the hydrogen bonds' overall strength [54].

### 3.6 Differential scanning calorimetry

**Fig. 4:**
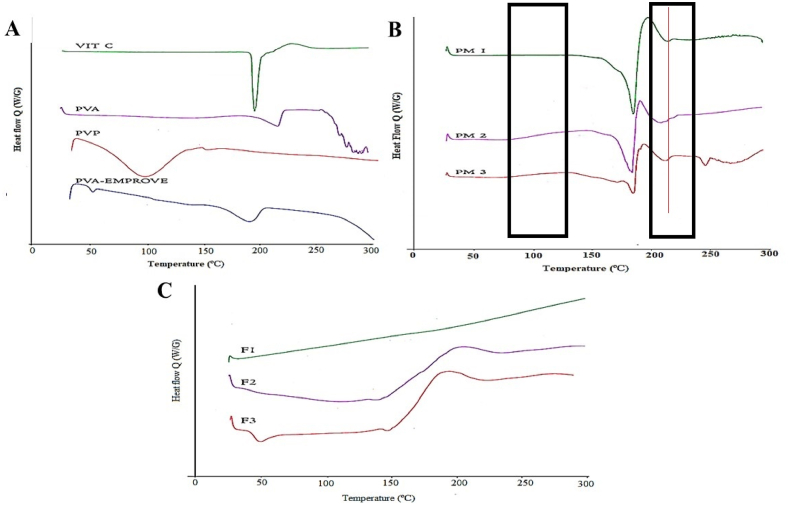
DSC thermograms of **(A)** VIT C, PVA, PVP, and PVA 5–88, **(B)** physical mixtures of F1-F3 MNs, and **(C)** F1-F3 VIT C MNs.

The DSC thermograms of pure VIT C, PVP, PVA, PVA 5–88, and F1-F3 MNs and their corresponding physical mixtures (PM1-PM3) are illustrated in Fig. 4. The DSC thermogram of VIT C (Fig. 4A) showed an endothermic peak at 194.80 °C, corresponding to its melting point. Additionally, an exothermic peak was observed at 227.95 °C, corresponding to the thermal degradation of VIT C [55]. The DSC thermograms of pure PVA and PVA 5–88 (Fig. 4A) showed endothermic peaks at 219.40 and 190.0 °C, respectively, corresponding to the melting points of the polymers, in agreement with [56,57]. Meanwhile, the DSC thermogram of pure PVP (Fig. 4A) did not reveal any typical endothermic or exothermic peak. However, it exhibits a broad peak around 100 °C, corresponding to its glass transition temperature and confirming its amorphous nature [56].

The DSC thermograms of PM1-PM3, corresponding to F1-F3 MNs, respectively, showed endothermic and exothermic peaks at 184.24 and 210.21 °C for PM1, 182.87 and 195.93 °C for PM2, and 184.40 and 211.89 °C for PM3 (Fig. 4B). Those observed peaks corresponded to the endothermic and exothermic peaks of VIT C and were slightly lower than those of pure VIT C (194.80 and 227.95 °C). This might be related to the presence of the polymer, which acts as an impurity, lowering the melting point of VIT C [58]. The endothermic peaks of PVA in PM1 and PM3 appeared around 219.40 °C at low intensity, owing to the dilution effect of the polymer. This is because the concentration of VIT C in F1 and F3 is high. Additionally, the DSC thermograms of PM2 and PM3, composed of PVP and PVA/PVP, respectively, showed weak endothermic peaks around 100 °C, corresponding to the PVP polymer. While PM1 showed the absence of the PVP peak since F1 is composed of only PVA. The absence or the low intensity of the endothermic peaks in the PM1-PM3 is attributed to the dilution effect that occurs when the drug content is high, suggesting that there is no interaction between the components when they are physically mixed [59]. Furthermore, the three PMs exhibited the corresponding endothermic and exothermic peaks of VIT C at lower intensities, with PM1 displaying the highest intensity and PM3 displaying the lowest.

The DSC thermograms of F1-F3 MNs (Fig. 4C) demonstrated no significant peaks for VIT C, suggesting that VIT C is completely dissolved in the MNs matrices [55]. These results are in agreement with [60], which showed that the disappearance of the characteristic peaks of the loaded drugs inside *in situ* microgels was attributed to drug entrapment within the molten polymeric carrier.

### 3.7 Attenuated total reflectance-Fourier transform infrared (ATR-FTIR)

**Fig. 5:**
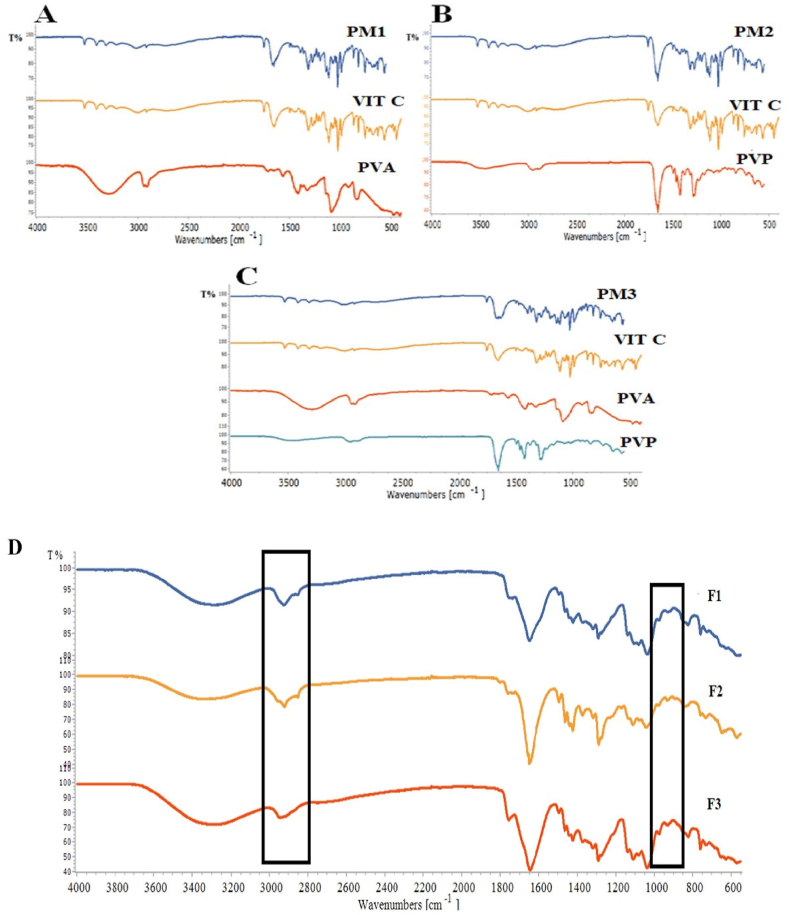
FTIR spectra of **(A**–**C)** pure VIT C, PVP, and PVA, and the physical mixtures (PM1-PM3) of F1-F3 MNs and **(D)** F1-F3 MNs.

The FTIR analysis was performed to identify the distinct peaks of materials that appear according to their structure, where any possible changes in the functional groups may indicate a chemical interaction between the materials used in the preparation [61]. The characteristic peaks of pure VIT C (Fig. 5A) at 1024, 1111, 1313, 1497, 1652, and 1753 cm^−1^ are assigned to the stretching and bending vibrations of C=O and OH groups [62]. Meanwhile, peaks at 3313, 3409, and 3525 cm^−1^ are assigned to the OH stretching, and those in the 2700-2916 cm-1 region are assigned to the C-H stretching. The C-H bending can be seen in a series of wavenumbers, including peaks at 869, 820, 754, 720, 678, and 1455 cm^−1^ [61]. The FTIR spectrum of PVA revealed peaks at 1092, 1422, and 2947 cm^−1^ (Fig. 5A). The peak at 1086 cm^−1^ is related to the carboxyl stretching band C–O and associated with the PVA's crystallinity. The C–H broad alkyl stretching band is also observed in a 2900–3000 cm-1 region. Because of the hydrophilic nature of PVA, the formation of hydrogen bonds is anticipated between PVA chains, with a hydrogen-bonded band appearing at a region of 3200–3570 cm^−1^. These results are in agreement with [63]. The FTIR spectrum of PVP exhibited major peaks around 1285 and 1652 cm^−1^ and weak peaks at 2885 and 2951 cm^−1^, corresponding to C-N, C=O, and C-H groups, respectively (Fig. 5B) [64].

In the FTIR spectra of the physical mixtures (PM1-PM3) (Fig. 5A–C), composed of VIT C, polymers, and stabilizing agents (EDTA/Meta), the functional groups of VIT C were exposed primarily. This includes three peaks observed at 3313, 3409, and 3525 cm^−1^ attributed to the OH stretching of VIT C. Additionally, the sharp peak observed at 1652 cm^−1^ can be attributed to the C=O stretching of VIT C. The distinct peaks of PVP and PVA in the PM1-PM3 spectra were not visible, masked by the sharp peaks of VIT C as they were mixed at ratios close to those used in MNs. However, the absence of any chemical shifts in the peaks of VIT C showed no chemical interaction between VIT C, polymers, and other excipients (EDTA/Meta).

The FTIR spectra of F1-F3 MNs (Fig. 5D) showed peaks at 1652 and 1758 cm^−1^, corresponding to VIT C. The crystalline VIT C in F2 is evidenced by the p-stacking peak at 900 cm^−1^ and the distinct carbonyl dimer at 2800 cm^−1^. However, the absence of both peaks in the F1 and F3 spectra confirmed that VIT C was dissolved in the MNs matrices. The absence of any shift in the C=O stretching or O-H stretching in F1-F3 spectra indicated the compatibility between VIT C, polymers, and other components.

### 3.8 *Ex vivo* permeation

**Fig. 6:**
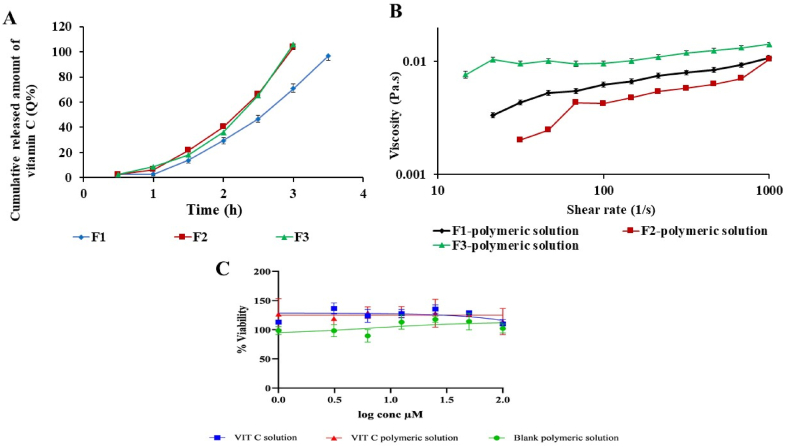
**(A)***Ex vivo* skin-permeation profiles of F1-F3 MNs, **(B)**Viscosity curves of VIT C polymeric solutions used to prepare F1-F3 MNs, and (**C**) %Viability of VIT C polymeric solution, VIT C solution (Free of polymers), and blank polymeric solution on the dermal fibroblast cell line evaluated by MTT assay.

Skin permeation studies were employed to explore the effect of F1-F3 MNs application on the transport of VIT C across rat skin. Fig. 6A shows the *ex vivo* skin-permeation profiles of VIT C delivered using F1-F3 MNs. The application of F1 resulted in skin permeation (%Q) of 96.6 ± 0.30 % of loaded VIT C after 3.5 h. Whereas skin permeation (%Q) of VIT C from F2 and F3 was 103.6 ± 2.04 and 105.8 ± 3.52 %, respectively, after 3 h.

Table 3 illustrates the skin permeation parameters of VIT C from F1-F3 MNs. F1 showed the highest cumulative amount of VIT C permeated through skin (Q/A) of 12346.3 ± 38.41 μg/cm^2^ in 3.5 h, followed by F2 and F3 of 12187.5 ± 239.88 and 6278.9 ± 170.70 μg/cm^2^, respectively, in 3 h. The enhanced permeation of VIT C across the skin is attributed to the synergistic effect of using the MNs combined with hydrophilic polymers (PVA and PVP). MNs can physically penetrate the SC barrier and favorably influence the transcutaneous transport of drugs. At the same time, the hydrophilic polymers fluidize the lipid layer of the SC, enhancing the permeation of VIT C [9,65].

**Table 3:** The permeation parameters of F1-F3 MNs obtained from the *ex vivo* skin-permeation profiles.

VIT C MNs	Q%	Q/A (μg/cm^2^)	J_ss_ (μg/cm^2^/h)	P × 10^−1^ (cm/h)
F1	96.6 ± 0.30	12346.3 ± 38.41	5262.2 ± 100.26	3.5 ± 0.06
F2	103.6 ± 2.04	12187.5 ± 239.88	5626.4 ± 99.14	4.0 ± 0.07
F3	105.8 ± 3.52	6278.9 ± 170.70	2841.3 ± 23.97	4.1 ± 0.03

The greatest steady-state flux (J_ss_) for VIT C was seen in F2 at 5626.4 ± 99.14 μg/cm^2^/h, followed by F1 and F3 at 5262.2 ± 100.26 and 2841.3 ± 23.97, respectively. Although F1 was loaded with the highest amount of VIT C, the observed difference in J_ss_ underlined the impact of the PVP polymer in F2 and F3 in enhancing the solubilization and drug partitioning, thereby influencing the release kinetics of VIT C across the SC [66]. This is because PVP is more hydrophilic than PVA and combines PVA/PVP [67]. Additionally, the permeability coefficient (P × 10^−2^) was higher for F2 (4.0 ± 0.07 cm/h) and F3 (4.1 ± 0.03 cm/h) compared to that of F1 (3.5 ± 0.06 cm/h). Therefore, F2 and F3 MNs fabricated with PVP or a combination of PVA/PVP could have better permeation than those fabricated with PVA only (F1 MNs).

### 3.9 Mechanism of VIT C release

The release mechanism of VIT C from F1-F3 MNs was investigated by fitting the permeation data into various mathematical models (zero-order, first-order, Higuchi, and Korsmeyer-Peppas) (Table 4). It was found that the release of VIT C from F1-F3 MNs perfectly followed the Korsmeyer-Peppas model with a regression line and high values of coefficients of determination (R^2^) of 0.9967, 0.9999, and 0.9993, respectively. The determined release exponent (n) for F1-F3 was >1, suggesting a super case-II transport mechanism. The super case-II release is attributed to the polymeric chain relaxation behavior brought about when MNs come in contact with the releasing medium, resulting in fast degradation and dissolution of the MNs [68]. Specifically, the hydrophilic polymers PVP and PVA would create channels that further enhance the dissolution of MNs [56,69].

**Table 4:** Calculated coefficient of determination (R^2^) and exponent of release mechanism (n) for F1-F3 MNs using the zero-order, first-order, Higuchi, and Korsmeyer-Peppas models.

VIT C MNs	Zero-Order	First-Order	Higuchi	Korsmeyer–Peppas	
	R^2^	R^2^	R^2^	R^2^	n
F1	0.9895	0.8688	0.9962	0.9967	>1
F2	0.9914	0.9955	0.9834	0.9999	>1
F3	0.9821	0.9978	0.9711	0.9993	>1

### 3.10 Viscosity studies

It was essential to investigate the viscosity of the polymeric solutions before pouring them into MN molds. This is because the viscosity of the polymeric solution might impact its ability to spread and fill the molds and determine the rate of VIT C release from MNs. Fig. 6B shows the viscosity curves of VIT C MNs polymeric solutions composed of 15 % PVA (F1), 40 % PVP (F2), and 15 % PVA:40 % PVP (2:1) (F3) used to prepare F1-F3 MNs, respectively. The viscosity of VIT C polymeric solutions followed a shear-thickening (dilatant flow) behavior, where the viscosity increased with increasing shear rate, in agreement with [70,71]. Additionally, the viscosity of the VIT C polymeric solutions slightly changed with shear rate and varied based on the type and concentration of the polymeric solutions. The viscosity of VIT C polymeric solutions was in the order F3 > F1 > F2, where the highest viscosity of F3 could be attributed to the high molecular weight and molar ratio of PVA and PVP, in agreement with [72]. The difference in the viscosity curves of the employed polymeric solutions was insignificant, with a *p*-value> 0.05.

### 3.11 Cytotoxicity of VIT C MNs

The MTT assay was carried out to evaluate the viability of the dermal fibroblast cells in the presence of VIT C polymeric solution, VIT C solution, and blank polymeric solution (Fig. 6C). It was found that VIT C polymeric solutions and blank polymeric solutions showed no toxicity at all concentrations, indicating that VIT C polymeric solutions are biocompatible and nontoxic to dermal cells. VIT C is known for its antioxidant activity, which neutralizes reactive oxygen species (ROS) in cells, causing more cellular growth in cells *in vitro,* where the freshly prepared VIT C solution and the VIT C polymeric solution increased cell viability [73].

### 3.12 Stability studies

**Fig. 7:**
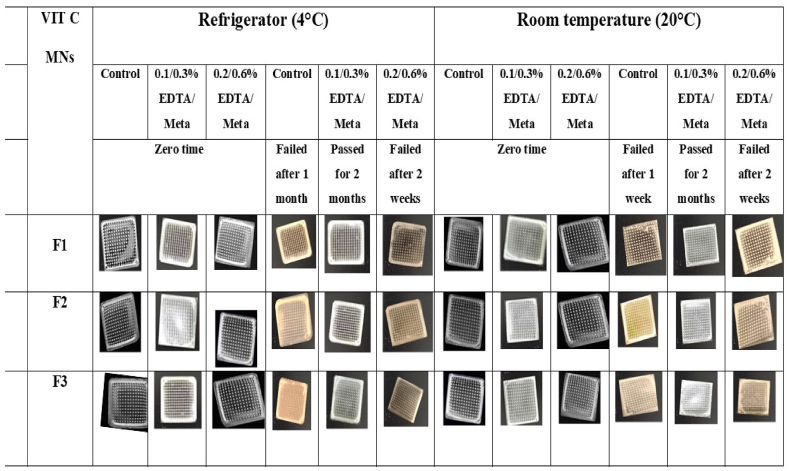
Color observation of F1-F3 MNs without stabilizing agents (control) and with 0.1/0.3 and 0.2/0.6 % EDTA/Meta after storage in the refrigerator (4 °C) and at room temperature (20 °C), observed under a USB digital microscope (Skybasic 50X-1000X) connected to a computer.

To determine the optimal VIT C MNs and the concentration of the stabilizing system required to maintain the physiochemical properties of VIT C, F1-F3 were subjected to stability studies at 4 and 20 °C for two months. The MNs were examined for morphology, mechanical properties, VIT C content, and dissolution rate (Fig. 7 and Table 5).

**Table 5:** Stability studies of VIT C MNs (F1-F3) without stabilizing agents (control) and with 0.1/0.3 and 0.2/0.6 % EDTA/Meta, tested for dissolution rate and VIT C content (mean ± SD).

Time	VIT C MNs	Stabilizers EDTA/Meta (% w/v)	Storage conditions			
			Refrigerator (4 °C)		Room temperature (20 °C)	
			Dissolution rate (min)	VIT C content (%)	Dissolution rate (min)	VIT C content (%)
**0**	**F1**	**Control**	20–30	89.85 ± 3.98	20–30	89.61 ± 3.95
		**0.1/0.3**	20–30	90.68 ± 3.63	20–30	90.93 ± 3.90
		**0.2/0.6**	20–30	90.09 ± 3.88	20–30	90.79 ± 4.13
	**F2**	**Control**	20–30	88.33 ± 3.26	20–30	88.24 ± 2.84
		**0.1/0.3**	20–30	88.61 ± 3.01	20–30	88.84 ± 3.04
		**0.2/0.6**	20–30	88.27 ± 3.01	20–30	88.55 ± 3.05
	**F3**	**Control**	20–30	87.05 ± 2.94	20–30	87.93 ± 3.24
		**0.1/0.3**	20–30	89.61 ± 1.37	20–30	89.98 ± 1.47
		**0.2/0.6**	20–30	89.00 ± 2.06	20–30	89.38 ± 1.17
**1**st **week**	**F1**	**Control**	30	85.05 ± 4.98	Failed in morphology and excluded from further study	
		**0.1/0.3**	30	86.05 ± 4.43	30	80.31 ± 1.59
		**0.2/0.6**	30	85.62 ± 4.71	30	78.47 ± 0.84
	**F2**	**Control**	30	84.41 ± 2.71	Failed in morphology and excluded from further study	
		**0.1/0.3**	30	85.82 ± 2.69	30	74.98 ± 0.57
		**0.2/0.6**	30	84.64 ± 2.92	30	71.67 ± 0.77
	**F3**	**Control**	30	84.03 ± 3.63	Failed in morphology and excluded from further study	
		**0.1/0.3**	30	85.99 ± 2.74	30	77.33 ± 4.02
		**0.2/0.6**	30	84.58 ± 3.04	30	74.27 ± 1.66
**2**nd **week**	**F1**	**Control**	40	81.64 ± 6.48	–	
		**0.1/0.3**	35	85.75 ± 4.59	30	79.23 ± 2.32
		**0.2/0.6**	Failed in morphology and excluded from further study		Failed in morphology and excluded from further study	
	**F2**	**Control**	40	65.77 ± 5.72	–	
		**0.1/0.3**	35	82.93 ± 2.02	30	70.65 ± 1.42
		**0.2/0.6**	Failed in morphology and excluded from further study		Failed in morphology and excluded from further study	
	**F3**	**Control**	40	69.41 ± 7.18	–	
		**0.1/0.3**	35	80.93 ± 3.33	30	71.36 ± 0.19
		**0.2/0.6**	Failed in morphology and excluded from further study		Failed in morphology and excluded from further study	
**1**st **month**	**F1**	**Control**	Failed in morphology and excluded from further study		–	
		**0.1/0.3**	35	82.95 ± 0.51	40	77.73 ± 0.09
	**F2**	**Control**	Failed in morphology and excluded from further study		–	
		**0.1/0.3**	35	76.17 ± 2.02	40	65.35 ± 1.53
	**F3**	**Control**	Failed in morphology and excluded from further study		–	
		**0.1/0.3**	35	77.73 ± 0.09	40	67.18 ± 1.08
**2**nd **month**	**F1**	**0.1/0.3**	35	80.45 ± 1.21	40–45	61.14 ± 0.18
	**F2**	**0.1/0.3**	35	70.02 ± 0.75	40–45	53.61 ± 2.78
	**F3**	**0.1/0.3**	35	74.49 ± 2.65	40–45	55.83 ± 0.88

F1-F3 were prepared without a stabilizing system (control group) and with a stabilizing system of EDTA/Meta at concentrations of 0.1/0.3 and 0.2/0.6 %. When observed under a light microscope, MNs showed a uniform and sharp pyramidal tip shape. The height and width of MNs were 601.5 ± 7.4 and 252 ± 2.0 μm, respectively, with MNs baseplates of acceptable mechanical tolerance and 20–30 min dissolution rates. At 4 °C, the initial content of VIT C for F1-F3 (control) was 89.85 ± 3.98, 88.33 ± 3.26, and 87.05 ± 2.94 %, respectively. Whereas F1-F3 containing 0.1/0.3 EDTA/Meta, the content of VIT C was 90.68 ± 3.63, 88.61 ± 3.01, and 89.61 ± 1.37 %, respectively. For F1-F3 containing 0.2/0.6 % EDTA/Meta, VIT C content was 90.09 ± 3.88, 88.27 ± 3.01, and 89.00 ± 2.06 %, respectively. At 20 °C, the content of VIT C for F1-F3 (control) was 89.61 ± 3.95, 88.24 ± 2.84, and 87.93 ± 3.24 %, respectively. Whereas F1-F3 containing 0.1/0.3 EDTA/Meta, the content of VIT was 90.93 ± 3.90, 88.84 ± 3.04, and 89.98 ± 1.47 %, respectively. For F1-F3 containing 0.2/0.6 % EDTA/Meta, the content of VIT C was 90.79 ± 4.13, 88.55 ± 3.05, and 89.38 ± 1.17 %, respectively.

After one week of storage at 4 °C, F1-F3 (control) and F1-F3 containing 0.1/0.3 and 0.2/0.6 % EDTA/Meta showed no change in color or appearance. Whereas a breakage in needles and a color change were observed for F1-F3 (control) when stored at 20 °C; hence, these MNs were excluded from further characterization. Contrarily, F1-F3 containing 0.1/0.3 and 0.2/0.6 % EDTA/Meta showed no change in color. The dissolution rates of all types of F1-F3 were comparable to those obtained initially after 30 min. VIT C content was reduced in all kinds of F1-F3 MNs, which was more notable at 20 °C than at 4 °C. For example, the content of VIT C at 4 and 20 °C was 85.05 ± 4.98 vs. 73.36 ± 0.75 % for F1 (control), 84.41 ± 2.71 vs. 69.86 ± 0.89 % for F2 (control), and 84.03 ± 3.63 vs. 70.62 ± 1.76 % for F3 (control). A similar reduction pattern was also noted in F1-F3 containing 0.1/0.3 and 0.2/0.6 % EDTA/Meta.

After two weeks of storage at 4 °C, F1-F3 (control) and F1-F3 (0.1/0.3 % EDTA/Meta) maintained their shape and mechanical strength. The dissolution rate of MNs was slightly increased to 35–40 min. The reduction of VIT C content was higher in F1-F3 (control) than in F1-F3 (0.1/0.3 % EDTA/Meta). For instance, the content of VIT C was 65.77 ± 5.72 vs. 82.93 ± 2.02 % for F2 (control) and F2 (0.1/0.3 % EDTA/Meta), respectively, and 65.77 ± 5.72 vs. 82.93 ± 2.02 % for F3 (control) and F3 (0.1/0.3 % EDTA/Meta), respectively. Moreover, after two weeks of storage at 20 °C, F1-F3 (0.1/0.3 % EDTA/Meta) showed no change in color and maintained their shape and mechanical strength. The dissolution rate for F1-F3 (0.1/0.3 % EDTA/Meta) was 30 min. The content of VIT C for F1-F3 (0.1/0.3 % EDTA/Meta) was 79.23 ± 2.32, 70.65 ± 1.42, and 71.36 ± 0.19 %, respectively. Contrarily, the needles of F1-F3 (0.2/0.6 % EDTA/Meta), stored at 4 and 20 °C, were broken upon applying mechanical force and displayed a color change to yellow due to the oxidation of VIT C [74]; hence, they excluded from further characterization. Therefore, stability studies were only conducted for F1-F3 (control) and those containing 0.1/0.3 % EDTA/Meta at 4 °C and for F1-F3 (0.1/0.3 % EDTA/Meta) at 20 °C.

After the first month of storage at 4 °C, F1-F3 (control) failed the morphological examination, displaying complete breakage of needles and a color change. Therefore, only F1-F3 (0.1/0.3 % EDTA/Meta) maintained their shape and color after the second month of storage at 4 and 20 °C. The dissolution rate of F1-F3 (0.1/0.3 % EDTA/Meta) decreased, where MNs completely dissolved after 35 min when stored at 4 °C and after 40–45 min when stored at 20 °C. The content of VIT C in F1-F3 (0.1/0.3 % EDTA/Meta) dramatically reduced at 4 and 20 °C. However, this reduction was lower at 4 °C than at 20 °C (80.45 ± 1.2 vs. 61.14 ± 0.18 %, 70.02 ± 0.75 vs. 53.61 2.78 %, and 74.49 ± 2.65 vs. 55.83 ± 0.88 % for F1-F3, respectively).

The results showed that loading VIT C into MNs with a stabilizing system of 0.1/0.3 % EDTA/Meta increased the physical and chemical stability of VIT C and maintained the shape and mechanical properties of MNs, particularly at 4 °C. Our findings are aligned with those of Maia et al. [75], who showed that the antioxidants Meta and glutathione increased the stability of VIT C in an oil-in-water (O/W) emulsion at 5 and 24 °C. However, the rate of degradation of VIT C was accelerated at 40 °C, even in the presence of the antioxidants. Hence, this demonstrates the need for a stabilizing system for VIT C formulations. The only contradiction was that Maia et al. [75] showed comparable reduction profiles of VIT C in O/W emulsions when stored at 5 and 24 °C. In contrast, our results demonstrated that the content of VIT C in all types of MNs was higher when stored at 4 °C than at 20 °C.

The mechanism by which EDTA/Meta interacts with VIT C might be complex and not fully understood. Huang et al. [76] showed that when the concentration of EDTA increased, the preservation rate of VIT C in acerola cherry pulp did not change significantly. This might be attributed to the fact that the effect of EDTA under acidic conditions is not as significant as under neutral conditions, where the acidic pH limits the impact of EDTA. Meta enhances the stability of VIT C by preventing its oxidation [12]. However, studies have shown that even with the addition of Meta, the degradation of VIT C may occur over time due to its high instability [11]. Hence, future studies are still warranted to pinpoint how the concentration of EDTA/Meta impacts the stability of VIT C MNs.

## 4 Conclusion

This work aimed to improve the dermal delivery of VIT C by incorporating it into dissolving MNs. The polymeric solutions employed in developing VIT C MNs resulted in a notable improvement in several factors, including the shape of needles, mechanical strength, dissolution rate, VIT C content, *ex vivo* permeation, and stability. Additionally, the present study addressed the crucial issue of stabilizing VIT C in delivery formulations by utilizing a stabilizing system of Meta/EDTA at an optimal ratio of 0.1/0.3 %. This stabilizing system was able to preserve the color, mechanical properties, and short-term chemical stability of VIT C MNs to penetrate the skin and deliver their cargo successfully. This study holds promise for advancing the dermal delivery of VIT C and other anti-aging agents, especially in the context of stability over prolonged periods, by loading them into MNs and using a stabilizing system. Finally, because the polymers used in fabricating the dissolving MNs (PVA and PVP) are less expensive when compared to the materials used in hollow or metal MNs, VIT C dissolving MNs could be more affordable than other types of MNs. In addition, PVA and PVP are FDA-approved for biomedical applications, non-toxic, and biocompatible with skin [50]. Moreover, the findings of the stability study indicated that loading VIT C into dissolving MNs with a stabilizing system of 0.1/0.3 % EDTA/Meta improved the physicochemical stability of VIT C and preserved the mechanical characteristics and shape of MNs, particularly when stored at 4 °C for two months. Concerning the mass production and the use protocols of VIT C MNs, future studies will focus on the scaling up the MNs’ patch size needed for successful human dosing and mass production.

## Ethics approval

The Research Ethics Committee at Al-Zaytoonah University of Jordan reviewed and approved this study with the approval number: (IRB # 01/10/2022–2023), dated [October 1, 2023].

## Funding

This study was funded by the 10.13039/501100021772Deanship of Scientific Research and Innovation at 10.13039/501100018786Al-Zaytoonah University of Jordan, grant # 13/08/2021-2022.

## Data availability

Data can be obtained from the corresponding author upon request.

## CRediT authorship contribution statement

**Rania Hamed:** Writing – review & editing, Resources, Project administration, Methodology, Investigation, Funding acquisition, Conceptualization. **Amani D. AbuKwiak:** Writing – original draft, Methodology, Investigation, Formal analysis. **Rafa Aburayya:** Writing – original draft, Methodology, Investigation, Formal analysis. **Ahlam Zaid Alkilani:** Writing – review & editing, Investigation, Conceptualization. **Lama Hamadneh:** Methodology. **Mais Naser:** Methodology, Investigation. **Yasmeen Al-Adhami:** Methodology, Investigation. **Ala A. Alhusban:** Methodology.

## Declaration of competing interest

The authors declare that they have no known competing financial interests or personal relationships that could have appeared to influence the work reported in this paper.
